# Comparative Evaluation of Platelet Indices in Clinical and Culture-Positive Neonatal Sepsis: A Prospective Observational Study

**DOI:** 10.7759/cureus.100485

**Published:** 2025-12-31

**Authors:** Pallavi Shaw, Ansser Vahab, Avinash L Sangle, Mohd Saeed Siddiqui, Mohammad Haseeb, Madhuri B Engade, Sachin Subhash Dhule

**Affiliations:** 1 Department of Pediatrics, Mahatma Gandhi Mission's (MGM) Medical College and Hospital, a Constituent Unit of MGM Institute of Health Sciences, Aurangabad, IND; 2 Department of Pediatrics and Neonatology, Mahatma Gandhi Mission's (MGM) Medical College and Hospital, a Constituent Unit of MGM Institute of Health Sciences, Aurangabad, IND

**Keywords:** culture-proven neonatal sepsis, mean platelet volume (mpv), neonatal septicemia, platelet distribution width neonatal sepsis, platelet indices

## Abstract

Background: Neonatal sepsis is among the significant causes of morbidity and mortality, especially in low- and middle-income countries. Blood culture, the diagnostic gold standard, is limited by delayed results and low sensitivity. Mean platelet volume (MPV) and platelet distribution width (PDW) may offer early, accessible biomarkers.

Objective: The objective of this study is to evaluate the diagnostic utility of platelet indices, i.e., MPV and PDW, in clinical and culture-positive neonatal sepsis.

Methods: A prospective observational study was conducted on 170 neonates with suspected sepsis admitted to the NICU at MGM Hospital, Aurangabad. Participants were categorized into clinical (n = 88) and culture-positive (n = 82) sepsis groups. Platelet parameters were analyzed.

Results: Of the 170 neonates enrolled, 82 (48.2%) had culture-positive sepsis, while 88 (51.8%) were categorized as clinical sepsis. MPV and PDW were significantly higher in both the culture-positive group and the clinical sepsis group.

Conclusion: MPV and PDW are significantly elevated in both the culture-confirmed neonatal sepsis and the clinical sepsis group. Platelet indices may serve as supportive diagnostic markers in resource-limited settings. Their integration with clinical and laboratory findings could enhance early diagnosis.

## Introduction

Neonatal sepsis, a leading cause of neonatal morbidity and mortality, has a variable clinical presentation, and the current diagnostic tools have limitations that make its timely diagnosis a significant challenge [1,2]. Blood culture, the current diagnostic gold standard, has a delayed turnaround time, with a low sensitivity in early-onset cases, and may give false negative results due to prior antibiotic administration or erratic sample collection [3]. Recent advances in automated culture systems have improved detection, but recovery rates remain highly dependent on sample volume and pre-analytic factors [4,5]. These constraints are especially problematic in resource-limited settings, where timely and reliable laboratory support may be lacking [1].

There is growing interest in identifying rapid, cost-effective, and easily accessible surrogate markers for early detection of sepsis [6]. Among hematologic markers, platelet indices, i.e., mean platelet volume (MPV), platelet distribution width (PDW), and plateletcrit (PCT), have emerged as potentially valuable tools [7,8]. The MPV and PDW are believed to reflect platelet activation and consumption, processes that are triggered by systemic inflammatory responses seen in sepsis [9]. Recent observational research data and meta-analyses have suggested that elevated MPV and PDW may correlate with sepsis, especially in preterm neonates [9,10].

However, the diagnostic utility of these indices remains unclear due to significant variability in findings across different populations and clinical settings. Some studies report statistically significant differences in platelet indices between septic and non-septic neonates, while others find these associations to be weak or non-specific [11,12]. Also, differentiation between culture-negative but symptomatic cases (clinical sepsis) and culture-positive sepsis is essential, as these may have different management implications [13].

This study was therefore conducted to evaluate the diagnostic significance of platelet indices MPV and PDW in clinical and culture-positive neonatal sepsis.

## Materials and methods

This prospective observational study was conducted in the Neonatal Intensive Care Unit (NICU) of MGM Hospital, Aurangabad, Maharashtra, from May 2023 to December 2024. The study was approved by the Institutional Ethical Committee prior to initiation. The sample size was limited by the time duration of the study.

Neonates aged less than 28 days who presented with clinical signs and symptoms suggestive of sepsis such as poor feeding, lethargy, tachypnoea, hypothermia, hypoglycemia, or convulsions were considered eligible for inclusion. Only those neonates whose parents provided written informed consent were enrolled in the study.

Neonates with congenital anomalies involving the gastrointestinal, respiratory, cardiovascular, or central nervous systems were excluded. Additional exclusion criteria included inborn errors of metabolism and prior exposure to antibiotic therapy before hospital admission.

After screening for eligibility and obtaining informed consent from the parents or legal guardians, venous blood samples were collected under strict aseptic precautions. Two milliliters of blood were drawn into EDTA vials for hematological analysis using an Advia 2120 analyzer (Siemens Medical Healthcare Diagnostics, Tarrytown, USA), which included platelet count, MPV, PDW, and C-reactive protein (CRP). Another four milliliters of venous blood were collected in two blood culture bottles and processed using the BACT/ALERT system, following optimized pediatric sampling protocols to improve recovery and reduce time-to-positivity.

All enrolled neonates were empirically started on broad-spectrum antibiotics, including cephalosporins and penicillin, and provided with supportive care as needed. Based on clinical features, hematological parameters, and microbiological test results, the neonates were categorized into two diagnostic groups: (i) Culture-proven sepsis: Neonates with positive blood culture results and compatible clinical or laboratory findings and (ii) Clinical sepsis: Neonates with clinical signs suggestive of sepsis, as per the WHO IMCI criteria, despite negative blood culture results.

Neonates diagnosed with culture-positive sepsis were treated with targeted antibiotic therapy based on sensitivity reports for a total duration of 14 days. Repeat cultures were obtained after the treatment period. If the neonate demonstrated clinical improvement and a negative repeat culture, they were discharged with instructions to return for follow-up after one week. Neonates with clinical sepsis continued empirical antibiotic therapy for five days, during which they were monitored daily for clinical and hematological improvement. If recovery was observed by the fifth day, the neonate was discharged and scheduled for a follow-up visit after one week. If laboratory abnormalities persisted, including deranged platelet indices or elevated CRP levels, a repeat blood culture was obtained. In such cases, if the culture turned positive, the antibiotic regimen was adjusted accordingly, and treatment was continued for 14 days until both clinical and laboratory recovery was confirmed.

All collected data were entered into Microsoft Excel and analyzed using IBM SPSS Statistics for Windows, Version 24 (Released 2016; IBM Corp., Armonk, New York, United States). Descriptive statistics, including mean and standard deviation, were used to summarize continuous variables, whereas categorical variables were expressed as proportions or percentages. The Pearson correlation coefficient was used to assess relationships between platelet indices and sepsis. A p-value of less than 0.05 was considered statistically significant.

## Results

A total of 170 neonates were enrolled in the study. Among these, 87 (51.2%) were male and 83 (48.8%) were female, indicating a slight male predominance. Most neonates (63.5%) presented between four and 21 days of life. The mean birth weight was 2.51 ± 0.57 kg. Table 1 shows the comparison of mean body weight among study groups.

**Table 1:** Body weight in clinical sepsis and culture-positive sepsis groups

Body Weight (kg)	Clinical Sepsis	Culture-Positive Sepsis	p-value (Unpaired t-test)
	Mean ± SD	Mean ± SD	
	2.51 ± 0.57	2.48 ± 0.59	0.74

Among the 170 neonates, 82 (48.2%) were diagnosed with culture-positive sepsis, while 88 (51.8%) had clinical sepsis. Both the MPV and PDW were significantly higher in the culture-positive sepsis group and the clinical sepsis group. Microbiological analysis revealed a predominance of Gram-negative organisms, particularly Klebsiella pneumoniae and Enterobacter species, consistent with regional neonatal infection trends.

Table 2 shows the platelet count in clinical sepsis and culture-positive neonatal sepsis groups. The platelet count was lower on Day 5 as compared to Day 1 in both clinical sepsis and culture-positive sepsis. There was statistically no significant difference between the groups.

**Table 2:** Platelet count in clinical sepsis and culture-positive sepsis groups

Platelet Count (per microliter of blood)	Clinical Sepsis (n = 82)	Culture-Positive Sepsis (n = 88)	t-value	p-value (Unpaired t test)
	Mean ± SD	Mean ± SD		
Day 1	215394.49 ± 95658.24	220127.49 ± 100476.08	0.31	0.75
Day 3	218305.50 ± 135347.22	212044.92 ± 106897.92	0.34	0.73
Day 5	200818.18 ± 117594.57	214178.45 ± 122015.56	0.73	0.47

Table 3 shows the MPV in clinical sepsis and culture-positive neonatal sepsis groups. The MPV showed a rising trend from day 1 to day 5 in both the study groups. The MPV was higher than the normal reference range in both the study groups.

**Table 3:** Mean platelet volume in clinical sepsis and culture-positive sepsis groups

Mean Platelet Volume (femtoliters)	Clinical Sepsis (n = 82)	Culture-Positive Sepsis (n = 88)	t value	p-value (Unpaired t-test)
	Mean ± SD	Mean ± SD		
Day 1	7.35 ± 0.85	7.26 ± 0.88	0.68	0.5
Day 3	7.48 ± 1.03	7.42 ± 1.07	0.37	0.71
Day 5	10.88 ± 17.16	7.69 ± 1.24	1.74	0.08

**Figure 1:**
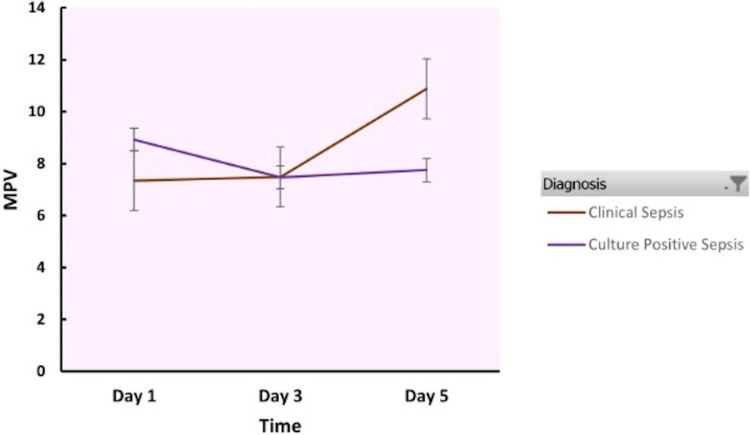
Mean platelet volume in clinical sepsis and culture-positive sepsis MPV: Mean platelet volume

Figure 1 depicts the MPV changes over the days in clinical sepsis and culture-positive neonatal sepsis groups.

Table 4 shows the PDW in clinical sepsis and culture-positive neonatal sepsis groups. The PDW was higher than the normal reference range in both the study groups.

**Table 4:** Platelet distribution width in clinical sepsis and culture-positive groups

Platelet Distribution Width (PDW)	Clinical Sepsis (n = 82)	Culture-Positive Sepsis (n = 88)	t value	p-value (Unpaired t-test)
	Mean ± SD	Mean ± SD		
Day 1	61.60 ± 12.81	61.70 ± 13.69	0.05	0.96
Day 3	61.18 ± 13.51	60.70 ± 12.91	0.24	0.81
Day5	59.10 ± 16.22	58.60 ± 17.27	0.19	0.85

**Figure 2:**
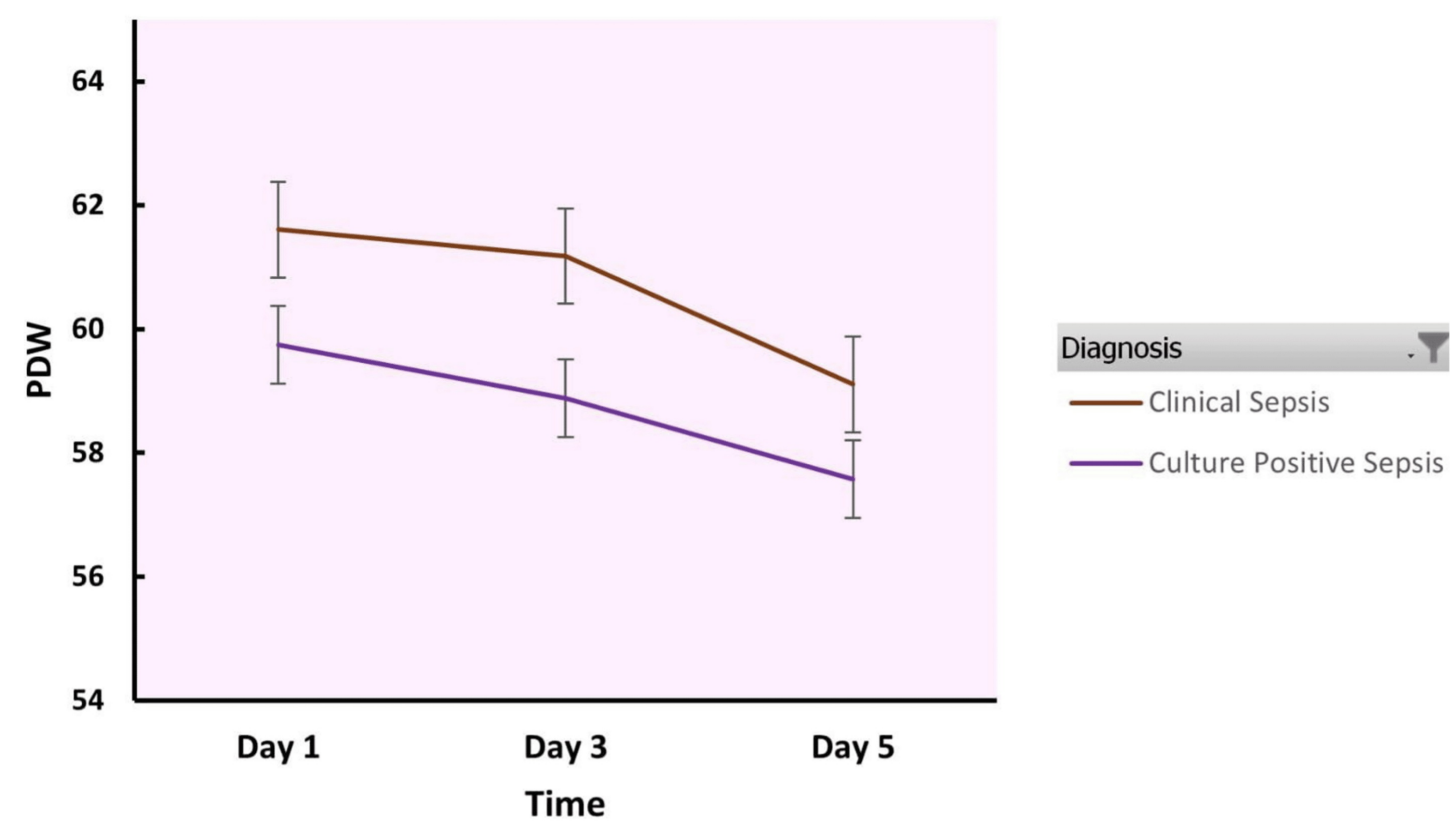
Platelet distribution width in clinical sepsis and culture-positive neonatal sepsis groups PDW: Platelet distribution width

Figure 2 depicts the PDW over the days in clinical sepsis and culture-positive neonatal sepsis groups.

Table 5 shows the CRP in clinical sepsis and culture-positive neonatal sepsis groups.

**Table 5:** C-reactive protein in clinical sepsis and culture-positive neonatal sepsis groups

C-Reactive Protein (CRP)	Clinical Sepsis (n = 82)	Culture Positive Sepsis (n = 88)	t value	p-value (Unpaired t-test)
	Mean ± SD	Mean ± SD		
Day 1	34.43 ± 28.57	41.86 ± 31.18	1.62	0.11
Day 3	45.32 ± 30.49	47.11 ± 29.58	0.39	0.7
Day5	52.35 ± 37.99	53.10 ± 37.92	0.13	0.90

The study results indicate that the MPV and PDW were higher in both clinical sepsis and culture-positive neonatal sepsis groups as compared to the normal reference values although there was no statistically significant difference between the groups. The MPV showed a rising trend over days 1 to day 5. The CRP was also raised in both the groups.

## Discussion

The MPV and PDW were significantly raised in both culture-confirmed and clinical neonatal sepsis. The MPV was higher in the clinical sepsis group. Neonatal sepsis is associated with thrombocytopenia [7,10,11,14,15] and a rise in the PDW and MPV in earlier studies and reviews [7,11,16,17]. 

The MPV indicates the mean platelet volume derived from the histogram on the automated Coulter counters. The platelet count and the cytokine-dependent megakaryocyte ploidy regulate the platelet volume. When there is decreased platelet production, young platelets enter circulation, and they are bigger and more active, and there is an increase in MPV levels. Increased MPV signifies an increased diameter of the platelets, and it is clinically useful as a marker of the platelet production rate and platelet activation. The Toll-like receptors are expressed on platelets, which allows them to identify bacterial proteins during sepsis. The Toll-like receptors, TLR2 and TLR4, play a role in augmenting platelet activation and help the platelets to add to their function from a hemostatic regulator to an immune sentinel. Thrombopoietin, which regulates megakaryocyte production, has been reported to be high in cases of thrombocytopenia in neonatal sepsis. Furthermore, septic neonates upregulate Tpo production, leading to increased megakaryocytopoiesis and platelet release. The rise in circulating TPo levels in the face of low platelet counts has been reported, and it has been suggested that research studies should be undertaken to evaluate whether high TPo levels after the dose of recombinant TPo would restore the platelet numbers [16,18-20]. Akarsu et al. studied PDW changes in cases of sepsis in neonates. Using a cutoff value of PDW of > 16.8 as high, they reported that 72.1% of cases with neonatal sepsis had raised PDW [21]. Guclu et al. have reported that patients with severe sepsis and more than 18 % PDW levels had a higher risk of death [11].

Catal et al. observed a positive correlation of MPV with IL-6 and CRP. A value of 10.35 fL was reported as the MPV cut-off for sepsis with a 97.8% sensitivity and 78.7% specificity. MPV of 10.75 fL and above was described as the reference value in sepsis patients, possibly resulting in death at diagnosis (sensitivity - 95.2% and specificity - 84.9%) [22].

Our study observations are in line with available literature, which indicates that platelet count, MPV, and PDW and CRP may serve as supportive diagnostic markers in resource-limited settings [23-28].

The limitations of our study include the single-center design, lack of a control group for comparison, and time-limited sample size, which may limit generalizability. Further large-scale, multicenter studies are recommended to validate the diagnostic and prognostic utility of platelet indices in neonatal sepsis.

## Conclusions

The study results and the relevant literature indicate that the platelet indices described may have a role in the diagnosis of sepsis in neonates. While conventional blood culture remains the gold standard, the early alterations in platelet count, MPV, and PDW may be promising and cost-effective adjunct indicators for the early detection of neonatal sepsis. The study findings emphasize the importance of a multiparametric diagnostic approach that combines clinical assessment with dynamic monitoring of hematologic and biochemical markers.
